# Missed confirmatory HCV RNA testing in anti-HCV-reactive patients and the role of reflex testing in improving completion of the hepatitis C diagnostic care cascade: a laboratory-based retrospective study

**DOI:** 10.1186/s12879-026-13807-4

**Published:** 2026-06-19

**Authors:** Özgür Appak, Nilay Danis, Sükran Köse

**Affiliations:** 1 Division of Medical Virology, Department of Medical Microbiology, Dokuz Eylul University School of Medicine, Izmir, Türkiye; 2 Division of Gastroenterology, Department of Internal Medicine, Dokuz Eylul University School of Medicine, Izmir, Türkiye; 3 Department of Infectious Diseases and Clinical Microbiology, Dokuz Eylul University School of Medicine, Izmir, Türkiye

**Keywords:** Hepatitis C virus, Anti-HCV, HCV RNA, Reflex testing, Care cascade, Diagnostic stewardship

## Abstract

**Background:**

Completion of confirmatory HCV RNA testing after anti-HCV reactivity is a critical step in the hepatitis C diagnostic care cascade. However, missed confirmatory testing and repeated anti-HCV testing within routine hospital laboratory workflows remain insufficiently characterized. This study aimed to evaluate the frequency of missed confirmatory HCV RNA testing among anti-HCV-reactive patients, investigate the presence of active infection using archived serum samples, and assess workflow-related diagnostic gaps that could potentially be addressed through laboratory-based reflex testing strategies.

**Methods:**

This retrospective laboratory-based observational study included patients tested for anti-HCV antibodies at a tertiary-care university hospital between January 1 and December 31, 2025. Anti-HCV-reactive patients without confirmatory HCV RNA testing were identified, and retrospective HCV RNA testing was performed on available archived serum samples. Repeated anti-HCV testing patterns and clinic-based differences in confirmatory testing requests were also analyzed to evaluate potential diagnostic workflow gaps.

**Results:**

Among 27,984 anti-HCV tests performed in 23,833 unique patients, the anti-HCV seroprevalence rate was 1.28%. Confirmatory HCV RNA testing was not requested in 32.3% of anti-HCV-reactive patients. Retrospective testing of archived serum samples from 79 patients without initial confirmatory testing identified active infection in 3.8% of cases (3/79). Confirmatory testing rates were significantly higher in specialist clinics than in non-specialist clinics (95.0% vs. 71.0%, *p* < 0.001). Repeated anti-HCV testing was observed in 13.2% of patients, and no seroconversion was detected among individuals tested six or more times.

**Conclusions:**

Missed confirmatory HCV RNA testing represents an important gap in the diagnostic care cascade, particularly in non-specialist clinical settings. Laboratory-based reflex HCV RNA testing strategies may improve completion of confirmatory testing and reduce unnecessary repeat serological testing, supporting more effective diagnostic stewardship and contributing to hepatitis C elimination efforts.

**Clinical trial number:**

Not applicable.

**Supplementary Information:**

The online version contains supplementary material available at https://doi.org/10.1186/s12879-026-13807-4.

## Introduction

Hepatitis C virus (HCV) causes chronic hepatitis, cirrhosis, and hepatocellular carcinoma. Worldwide, 71 million people are infected with HCV, and 400,000 deaths occur each year. Although there are 1.5 million new cases annually, only 21–30% of these are diagnosed [1]. Approximately 70% of individuals infected with HCV develop chronic infection, leading to complications [2]. It is estimated that there are approximately 1–1.5 million chronic HCV patients in Türkiye. Current, large-scale, population-based data on the epidemiology of hepatitis C in our country are limited. While hospital-based studies report anti-HCV prevalence of 1.5–2.5%, the rate is 0–0.1% in healthy young populations. Overall, the prevalence is estimated to be slightly below 0.3%. Türkiye has national recommendations for HCV screening. According to the Turkish Hepatitis C Diagnosis and Treatment Guidelines, screening is recommended for high-risk populations, including people who inject drugs, hemodialysis patients, individuals with a history of blood transfusion or organ transplantation, and those with HIV/HBV co-infection. In addition, at least one anti-HCV test is recommended for all individuals presenting to healthcare institutions, even in the absence of known risk factors [3]. In routine tertiary-care hospital practice, anti-HCV testing is also commonly performed as part of preoperative evaluation, employee health screening, transplantation programs, and immunosuppressive treatment follow-up.

Today, short treatment durations and high response rates are achieved with direct-acting antivirals (DAAs) against HCV. Systematic reviews on the effectiveness of DAA drugs show that sustained virological response rates generally exceed 95% [4]. Therefore, early diagnosis of HCV and initiation of treatment are extremely important in terms of preventing morbidity, mortality, and transmission.

Serological methods (anti-HCV antibody testing, HCV core Ag detection, Ag + Ab combination assays) and molecular methods (real-time qPCR) are used to diagnose HCV. The current diagnostic algorithm for HCV begins with anti-HCV screening. If the result is reactive, in many clinical workflows, the individual is required to return to the clinic and provide a second blood sample for HCV RNA testing [5]. In low-prevalence populations, since the majority of screening test results are negative, low-cost anti-HCV antibody tests are used as the initial test. However, in many cases, physicians do not request a second blood sample, or patients do not return to the hospital. The turnaround time for test results ranges from 1 to 3 days for anti-HCV antibody tests and from 3 days to 2 weeks for HCV RNA tests [6]. It has been reported that confirmatory HCV RNA testing is not performed in a significant proportion of anti-HCV-reactive patients, which constitutes an important breakpoint in the HCV care cascade. For this reason, the concept of reflex testing has been proposed in HCV diagnosis to prevent missed diagnoses and delays. There are two types of reflex testing applications: laboratory-based and clinic-based. In both methods, patients may receive a diagnosis with a single hospital visit in many clinical settings. Laboratory-based reflex testing involves performing HCV RNA testing on the same blood sample from patients who test reactive for anti-HCV antibodies, whereas clinic-based reflex testing involves performing rapid anti-HCV antibody testing by the clinician and, if reactive, obtaining a second blood sample for HCV RNA testing [7]. Reflex testing has been shown to improve confirmatory testing uptake and shorten time to diagnosis and treatment initiation. However, real-world data evaluating both the lack of confirmatory HCV RNA testing and the patterns of repeated anti-HCV test requests within hospital-based laboratory workflows, particularly in relation to clinic-specific testing behavior and archived-sample RNA detection, remain limited.

At the Dokuz Eylül University Hospital Central Laboratory, a standard two-step diagnostic algorithm is used for HCV testing. The primary aim of this study was to evaluate the frequency of missed confirmatory HCV RNA testing among anti-HCV-reactive patients and to investigate the presence of active infection by retrospective HCV RNA testing of archived serum samples. The secondary aim was to analyze patterns of repeated anti-HCV testing and to assess the potential contribution of a laboratory-based reflex testing strategy to reducing diagnostic gaps and unnecessary repeat serology. By combining missed confirmatory testing, retrospective HCV RNA detection in archived samples, clinic-specific testing behavior, repeated anti-HCV testing patterns, and anti-HCV S/CO dynamics, this study provides a workflow-based assessment of where the HCV diagnostic cascade fails in routine hospital practice.

## Methods

This retrospective, laboratory-based observational study was conducted at the Central Laboratory of Dokuz Eylül University Hospital to evaluate missed confirmatory HCV RNA testing, repeated anti-HCV testing patterns, and the potential impact of reflex testing strategies within routine hospital diagnostic workflows. Serum samples with sufficient volume from patients who were found to be anti-HCV-reactive among those sent to the serology laboratory for anti-HCV antibody testing between January 1, 2025, and December 31, 2025, were included if they had no prior diagnosis of HCV infection and had not undergone confirmatory HCV RNA testing. A complete one-year study period was intentionally selected to capture routine anti-HCV testing practices across all clinical departments, minimize potential short-term fluctuations in testing behavior and referral patterns, and ensure an adequate sample size for evaluating confirmatory HCV RNA testing practices and missed opportunities in the HCV diagnostic care cascade. Among the 99 anti-HCV-reactive patients for whom confirmatory HCV RNA testing had not been requested initially, archived serum samples with sufficient volume were available for retrospective HCV RNA analysis in 79 patients, and HCV RNA testing was successfully performed in these samples. All available samples were stored at − 80 °C until analysis and processed after a single thaw cycle. Patients who had previously undergone HCV RNA testing were excluded from the study. Patient records were retrospectively evaluated using the hospital information management system, and the study was conducted using archived samples without any direct patient intervention.

To evaluate the potential impact of a laboratory-based reflex testing strategy, anti-HCV-reactive patients without an initial confirmatory HCV RNA request were identified, and available archived serum samples from these patients were retrospectively tested for HCV RNA. This approach allowed estimation of how many active infections could have been detected if confirmatory testing had been automatically performed on the same sample within the routine diagnostic workflow.

Anti-HCV antibody tests were performed using the chemiluminescent microparticle immunoassay (CMIA) method on the Abbott Alinity i (Abbott Diagnostics, USA). Results were evaluated according to the manufacturer’s recommended cut-off values, and samples with a signal-to-cut-off ratio (S/CO) ≥ 1.0 were considered reactive. Anti-HCV-reactive results were routinely verified by repeat testing using the same assay, and samples showing reactivity in two consecutive measurements were considered confirmed reactive and included in the analysis.

Nucleic acid extraction was performed using the Bio-speedy^®^ rapid nucleic acid extraction kit (Bioeksen, Türkiye) on the EXM 3000 (Zybio, China) automated extraction platform according to the manufacturer’s instructions. All archived serum samples were originally collected for routine clinical testing and reused for retrospective molecular analysis within the scope of the present study.

HCV RNA amplification and quantitative analysis were performed using the RealStar^®^ HCV RT-PCR Kit 2.0 RUO (Altona Diagnostics, Germany) on the Rotor-Gene Q real-time PCR platform (QIAGEN, Germany), in accordance with the manufacturer’s recommendations. Positive and negative controls, together with an internal control, were included in each run to ensure analytical validity and monitor extraction efficiency and PCR inhibition. According to the manufacturer’s specifications, the assay’s analytical limit of detection (LoD) is approximately 12 IU/mL for EDTA plasma samples. Although archived serum samples were used in this retrospective analysis, all procedures were performed in accordance with the manufacturer’s recommendations within the routine workflow of the institutional molecular diagnostics laboratory. These retrospective analyses were performed for research purposes only and did not influence clinical decision-making. Routine clinical confirmatory HCV RNA testing at our institution was performed using the Roche cobas^®^ platform and was independent of the retrospective HCV RNA analyses performed in this study using the RealStar^®^ HCV RT-PCR Kit 2.0 RUO (Altona Diagnostics).

Statistical analysis of the obtained data was performed using IBM SPSS Statistics version 22 (IBM Corp., Armonk, NY, USA). Descriptive variables were expressed as numbers and percentages. The distribution of continuous variables was assessed for normality using the Kolmogorov–Smirnov test. Normally distributed continuous variables were compared using Student’s t-test, whereas non-normally distributed variables were analyzed using the Mann–Whitney U test. Since age distribution was non-normal, age comparisons between anti-HCV-reactive and anti-HCV-negative groups were performed using the Mann–Whitney U test. Repeat anti-HCV testing patterns within the same study period were evaluated to determine the frequency and clinical context of repeat testing and to assess whether repeat testing was associated with the detection of new active infection. For subgroup analyses, specialist clinics were defined as hepatology and transplantation-related units directly involved in the diagnosis, follow-up, or management of chronic liver disease and viral hepatitis, whereas all remaining clinical departments were classified as non-specialist clinics. The relationship between anti-HCV S/CO levels and HCV RNA results was analyzed. The chi-square test or Fisher’s exact test, when necessary, was used to compare categorical variables. The statistical significance level was accepted as *p* < 0.05.

This study was approved by the Dokuz Eylul University Non-Interventional Research Ethics Committee (approval number: 2025/02–15, date: 15.01.2025) and was conducted in accordance with the Declaration of Helsinki. The requirement for informed consent was waived due to the retrospective design and use of archived anonymized laboratory data. The waiver of informed consent also covered the retrospective longitudinal evaluation of repeated routine laboratory measurements included in the study. The longitudinal analyses performed in this study were entirely retrospective and based solely on repeated routine laboratory measurements recorded within the hospital information management system during the predefined study period. No prospective follow-up, direct patient contact, additional blood sampling, or research-related intervention was performed for patients included in the longitudinal analyses. No change in routine clinical practice or diagnostic workflow was implemented during the study period. Physicians were not contacted regarding anti-HCV-reactive results, no additional EDTA samples were requested, and retrospective HCV RNA analyses were performed only after completion of the study period using archived residual serum samples.

## Results

During the study period, a total of 27,984 anti-HCV tests were performed in 23,833 unique patients. It was determined that repeat anti-HCV testing was requested in 13.2% (3,140/23,833) of the patients. The overall anti-HCV seroprevalence rate among unique patients was 1.28% (306/23,833).

The median age of anti-HCV-reactive patients was 65 years (IQR: 49.5–73.0), whereas the median age of anti-HCV-negative patients was 53 years (IQR: 34.0–68.0). Anti-HCV-reactive patients were significantly older than anti-HCV-negative patients (Mann–Whitney U test, *p* < 0.001). Among anti-HCV-reactive patients, 54.7% were female, and 45.3% were male.

The most frequent first anti-HCV test requests originated from the ophthalmology outpatient clinic (10.46%), employee health unit (7.07%), urology department (5.72%), and obstetrics and gynecology department (4.86%). These percentages represent only the most frequently requested clinical units and therefore do not reflect the complete distribution of anti-HCV test requests across all departments. Among anti-HCV-reactive patients, the most frequent clinical units requesting the initial anti-HCV test were the ophthalmology outpatient clinic (13.0%), hepatology outpatient clinic (9.8%), and general surgery/transplantation outpatient clinic (8.5%).

**Fig. 1:**
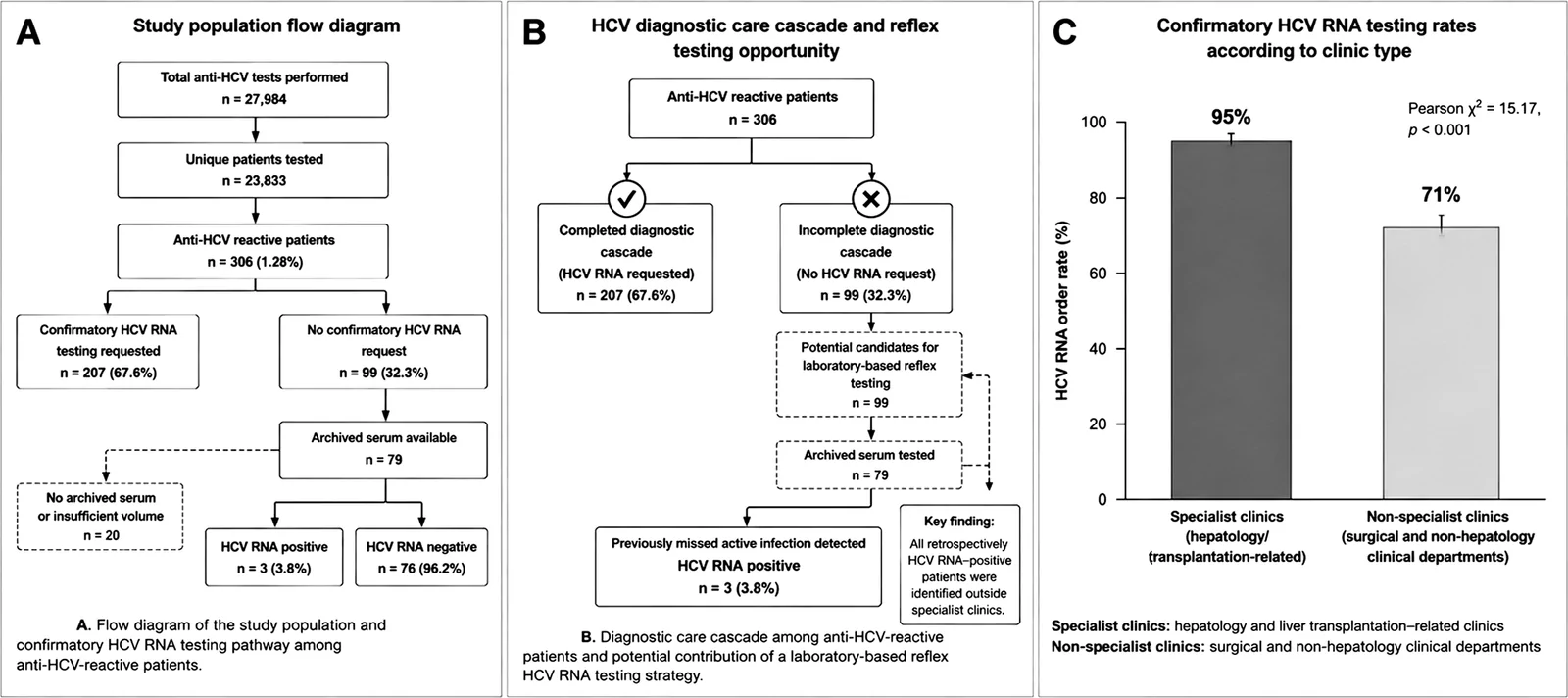
Study population flow, HCV diagnostic care cascade, and confirmatory testing patterns. **A**) Flow diagram of the study population. **B**) HCV diagnostic care cascade among anti-HCV-reactive patients. **C**) Comparison of confirmatory HCV RNA testing request rates between specialist (hepatology/transplantation-related) and non-specialist clinics

The overall study population flow, HCV diagnostic care cascade, and comparison of confirmatory HCV RNA testing rates between specialist and non-specialist clinics are presented in Fig. 1A and C.

Confirmatory HCV RNA testing was requested in 67.6% (207/306) of anti-HCV-reactive patients, whereas 32.3% (99/306) did not undergo confirmatory testing. Among patients with clinician-requested confirmatory HCV RNA testing, HCV RNA positivity was detected in 9 patients (4.3%; 9/207). Under a laboratory-based reflex testing model, these 99 patients would have been eligible for automatic HCV RNA testing using the same sample, eliminating the need for an additional clinical request or patient revisit.

Among the 99 patients without confirmatory HCV RNA testing, retrospective NAT analysis was feasible in 79 archived samples (Fig. 1A), and active infection was identified in 3 patients (3.8%). The clinical distribution of patients without confirmatory HCV RNA testing showed that the majority (96.0%) had their initial anti-HCV test requested outside hepatology or transplantation-related clinics. Only 4.0% originated from specialist clinics, suggesting more consistent confirmatory testing practices in these settings. Accordingly, when clinical units were grouped as specialist (hepatology/transplantation-related) and non-specialist clinics, the confirmatory HCV RNA testing request rate was significantly higher in specialist clinics (95.0% vs. 71.0%; Pearson χ²=15.17; *p* < 0.001).

Retrospective HCV RNA testing was performed in 79 patients with available archived serum samples, and HCV RNA positivity was detected in 3 patients (3.8%). All HCV RNA–positive patients were identified among individuals tested outside hepatology clinics. In all patients with HCV RNA positivity, anti-HCV S/CO values were markedly elevated (> 16). The distribution of anti-HCV S/CO values according to HCV RNA status is presented in Supplementary Figure S1. Overall, HCV RNA-positive patients tended to have higher anti-HCV S/CO values than HCV RNA-negative patients. The demographic and laboratory characteristics of HCV RNA-positive patients are presented in Table 1.

**Table 1:** Patients with retrospectively detected HCV RNA positivity

Patient ID	Age (years)	Sex	Requesting clinic	Anti-HCV S/CO index*	HCV RNA (Ct)	HCV RNA (IU/mL)	Initial RNA requested
Patient 1	70	Female	Ophthalmology outpatient clinic	17.42	21.44	873	No
Patient 2	83	Female	Coronary intensive care unit	16.19	21.99	591	No
Patient 3	71	Female	Ophthalmology outpatient clinic	20.92	23.15	259	No

* Anti-HCV positivity defined as S/CO ≥ 1.0

Ct: Cycle threshold; IU/mL: International units per milliliter

When the frequency of repeated anti-HCV testing during the study period was examined, it was observed that the majority of repeat tests consisted of patients tested twice (*n* = 2,484). This was followed by patients tested three times (*n* = 468), four times (*n* = 113), and five times (*n* = 45). The number of patients tested six or more times was 30, and the maximum number of repeat tests in this group was nine (Supplementary Table S1A). It was observed that patients in the high repeat-testing group were mainly those followed in immunosuppression and transplantation programs, including hematology, bone marrow transplantation, general surgery transplantation, and nephrology (Supplementary Table S1B). Notably, no seroconversion was detected among patients who underwent repeated anti-HCV testing six or more times during the study period, despite some individuals being tested up to nine times.

When longitudinal anti-HCV results were examined, a transition from negative to reactive was observed in five patients, and from reactive to negative in three patients. In patients who transitioned from negative to reactive, anti-HCV index values were mostly low and fluctuated during follow-up. Active infection was not detected in any of these patients with confirmatory HCV RNA testing. Similarly, in patients who showed a transition from reactive to negative, low-index reactivity and subsequent negativization during follow-up, this was considered consistent with borderline reactivity or possible false positivity (Table 2).

**Table 2:** Longitudinal anti-HCV serological transitions in patients with index variability during follow-up

Patient ID	Transition Type	Sequential Anti-HCV S/Co Results *	Clinical Comment	HCV RNA
Patient 1	Neg → Reac	Neg (0.64) → Neg (0.51) → Reac (2.14)	Low-index positivity; probable borderline seroconversion	Negative
Patient 2	Neg → Reac → Neg	Neg (0.10) → Reac (1.32) → Reac (1.33) → Neg (0.92) → Neg (0.22)	Typical borderline reactivity fluctuation	Negative
Patient 3	Neg → Reac → Neg	Neg (0.12) → Reac (6.48) → Neg (0.34)	Moderate-level positivity; possible true seroconversion	Negative
Patient 4	Neg → Reac	Neg (0.34) → Neg (0.30) → Reac (1.11)	Low-index positivity during transplant screening	Negative
Patient 5	Neg → Reac	Neg (0.25) → Reac (1.61)	Borderline positivity during employee health screening	Negative
Patient 6	Reac → Neg	Reac (1.14) → Neg (0.90)	Low-index positivity followed by negativization; borderline reactivity likely	Negative
Patient 7	Reac → Neg	Reac (1.11) → Reac (1.18) → Neg (0.92)	Fluctuating low-index reactivity	Negative
Patient 8	Reac → Neg	Reac (1.12) → Neg (0.41) → Neg (0.41) → Neg (0.35)	Persistent negativization after positivity; possible false positivity	Not available

* Anti-HCV positivity defined as S/CO ≥ 1.0

Neg: negative; Reac: reactive; S/CO: signal-to-cutoff ratio

## Discussion

In this study, we evaluated anti-HCV test ordering patterns and the use of confirmatory HCV RNA testing in a large hospital population and demonstrated an important gap in the hepatitis C diagnostic care cascade. Importantly, this study provides a workflow-based evaluation integrating missed confirmatory testing, archived-sample RNA detection, clinic-specific testing behavior, and repeated anti-HCV testing patterns within a single laboratory dataset. Despite current guideline recommendations, confirmatory HCV RNA testing was not requested in approximately one-third of patients with anti-HCV-reactive results. Retrospective analysis of archived serum samples from these patients revealed that a proportion of active infections could remain undiagnosed in routine clinical workflows without reflex testing strategies. In addition, the older mean age of anti-HCV-reactive patients in our study is consistent with epidemiological studies showing that HCV seroprevalence increases with age. Although some studies have reported higher anti-HCV prevalence in men [8], no significant difference between sexes was observed in our study. Although our study population does not represent a population-based sample, it included a large and heterogeneous tertiary-care hospital cohort comprising patients from routine outpatient care, employee health screening, surgical services, transplantation programs, and subspecialty clinics. Therefore, while the observed age distribution appears consistent with regional and national epidemiological trends, our findings should primarily be interpreted as reflecting real-world diagnostic practices within a tertiary-care hospital setting rather than directly representing the general population.

To achieve hepatitis C elimination targets, increasing screening rates alone is insufficient; completing confirmatory HCV RNA testing after anti-HCV reactivity is a critical step in the diagnostic care cascade. Previous studies across different healthcare settings have demonstrated that reflex testing strategies significantly increase confirmatory testing uptake and shorten time to diagnosis and treatment initiation [9–12]. In particular, reflex testing has been shown to reduce diagnostic delays, minimize patient loss between testing steps, and improve linkage to care, especially in non-specialist clinical settings [11–14]. In our institution, routine confirmatory HCV RNA testing is performed using an automated Roche cobas^®^ workflow. Therefore, delays in completion of the diagnostic cascade are primarily related to the need for an additional clinician request and, in some cases, a repeat patient visit or blood draw rather than assay-related turnaround time. Under a laboratory-based reflex testing strategy, confirmatory HCV RNA testing could be initiated using the same sample following anti-HCV reactivity, potentially reducing diagnostic delay by several days and improving completion of the diagnostic care cascade. In line with these observations, our findings suggest that laboratory-based reflex HCV RNA testing may represent a practical and scalable strategy to reduce workflow-related diagnostic gaps identified in our hospital population.

In our study, anti-HCV test requests were most frequently made by the ophthalmology outpatient clinic, the employee health unit, and surgical departments, suggesting that testing was often performed as part of routine screening rather than clinical suspicion. Notably, anti-HCV positivity rates in the employee health unit, urology, and obstetrics/gynecology clinics were lower than those observed in the remaining hospital departments, supporting the interpretation that testing in these settings was largely performed as part of routine screening rather than clinical suspicion. In contrast, ophthalmology demonstrated a positivity rate comparable to the overall hospital population. In contrast, confirmatory testing rates were substantially higher in specialist clinics such as hepatology and transplantation units. Notably, all patients with retrospectively detected HCV RNA positivity were identified among individuals tested outside hepatology clinics, indicating that missed confirmatory testing may lead to clinically relevant active infections being overlooked, particularly in non-specialist clinical settings. However, this finding should be interpreted with caution, as patients attending hepatology- and transplantation-related clinics may include individuals with pre-existing liver disease, prior HCV diagnosis, or transplant-related follow-up, potentially contributing to greater clinician awareness and adherence to confirmatory testing practices. In our institution, transplantation-related services primarily include liver, kidney, and bone marrow transplantation programs, where anti-HCV testing is frequently performed as part of routine pre-transplant evaluation and follow-up. These findings highlight the importance of diagnostic stewardship–based interventions targeting confirmatory testing practices beyond specialist settings [15].

Another important observation in our study was the frequent repetition of anti-HCV testing within the same year in a subset of patients. Notably, no seroconversion was detected among patients who underwent repeated testing six or more times, despite some individuals being tested up to nine times during the study period. These findings suggest that repeated anti-HCV testing may provide limited additional diagnostic yield in certain clinical settings, particularly among patients already under follow-up in immunosuppression or transplantation programs. From a laboratory stewardship perspective, this pattern indicates a potential area for optimization, as implementation of reflex confirmatory HCV RNA testing strategies may help reduce unnecessary repeat serological testing while improving completion of the diagnostic algorithm [16].

The performance of S/CO threshold values in anti-HCV assays for predicting active infection may vary across test platforms, population characteristics, and background prevalence. Previous studies have reported a wide range of proposed S/CO thresholds across different immunoassay systems, and no universal threshold has been identified that can reliably replace confirmatory HCV RNA testing [17, 18]. In our study, anti-HCV index values were markedly elevated (> 16) in all patients retrospectively found to be HCV RNA-positive. Although the number of cases was limited, this observation suggests that high-index reactivity may help prioritize confirmatory HCV RNA testing within reflex testing–based diagnostic workflows. In contrast, longitudinal fluctuations observed in low-index anti-HCV reactivity were not associated with detectable active infection and were considered consistent with borderline reactivity or possible false positivity.

Of note, patients demonstrating a reactive-to-non-reactive anti-HCV pattern (patients #6–#8 in Table 2) were not receiving documented immunosuppressive therapy and were not followed in transplantation- or hematology-related programs. Furthermore, all exhibited only low-index reactivity near the assay cut-off (S/CO range: 1.11–1.14), followed by subsequent non-reactive results and negative (or unavailable) HCV RNA testing, supporting the interpretation of borderline reactivity or analytical fluctuation rather than persistent infection. Because no detectable HCV RNA was identified in patients demonstrating longitudinal anti-HCV serological transitions, genotype analysis was not feasible. Therefore, no genotype-specific explanation for the observed serological fluctuations could be investigated.

Integration of automatic reflex HCV RNA testing algorithms into laboratory information systems may help standardize confirmatory testing, particularly for samples requested from non-specialist clinics. Such laboratory-based interventions may reduce patient loss in the diagnostic care cascade, support a more rational use of laboratory resources, and contribute to hepatitis C elimination strategies [13, 19]. These findings support the implementation of laboratory-based reflex testing algorithms as a feasible diagnostic stewardship intervention in routine hospital practice.

From a scientific perspective, this study provides a workflow-based evaluation integrating missed confirmatory HCV RNA testing, retrospective molecular assessment of archived serum samples, clinic-specific testing behavior, repeated anti-HCV testing patterns, and anti-HCV S/CO dynamics within a single tertiary-care laboratory dataset. Unlike previous reports primarily evaluating reflex testing implementation, our findings additionally demonstrate real-world missed active infections among anti-HCV-reactive patients who did not undergo clinician-requested confirmatory testing. Clinically, identification of non-specialist clinics as major points of attrition in the diagnostic care cascade highlights a practical target for diagnostic stewardship interventions. These findings support laboratory-based reflex HCV RNA testing as a feasible strategy to reduce missed diagnoses, unnecessary repeat serological testing, and delays in linkage to care.

This study has several limitations. First, the study has a single-center retrospective design, and the results may not be directly generalizable to different populations. Second, confirmatory HCV RNA analysis could be performed only in patients with sufficient archived serum samples, potentially introducing selection bias. Third, referral to treatment and access to antiviral therapy among patients with detectable HCV RNA could not be evaluated. Finally, because this was a retrospective laboratory-based study, the subsequent clinical management and treatment outcomes of patients with retrospectively detected HCV RNA positivity could not be assessed. In addition, confirmatory HCV RNA testing was performed using archived serum rather than plasma samples, which may represent a minor analytical limitation. In addition, the small number of retrospectively detected HCV RNA-positive cases limits the ability to draw conclusions regarding predictive thresholds for anti-HCV S/CO values.

In conclusion, this study provides a workflow-based evaluation of missed confirmatory HCV RNA testing within a large tertiary-care hospital setting and demonstrates that a substantial proportion of anti-HCV–reactive patients did not complete the diagnostic algorithm, particularly in non-specialist clinical units. Retrospective detection of active infection among patients without initial confirmatory testing highlights the potential clinical impact of these diagnostic gaps. By integrating archived-sample RNA detection, clinic-specific testing behavior, and repeated anti-HCV testing patterns within a single laboratory dataset, our findings support the implementation of laboratory-based reflex HCV RNA testing as a feasible diagnostic stewardship intervention. Such workflow-oriented strategies may improve completion of the hepatitis C diagnostic care cascade and contribute to ongoing hepatitis C elimination efforts in routine clinical practice.

## Supplementary Information

Below is the link to the electronic supplementary material.


Supplementary Material 1



Supplementary Material 2



Supplementary Material 3


## Data Availability

The datasets used and/or analyzed during the current study are available from the corresponding author on reasonable request.

## Abbreviations

HCV: Hepatitis C virus
DAA: Direct-acting antiviral
anti-HCV: Antibody to hepatitis C virus
HCV RNA: Hepatitis C virus ribonucleic acid
Ag: Antigen
Ab: Antibody
qPCR: Quantitative polymerase chain reaction
CMIA: Chemiluminescent microparticle immunoassay
S/CO: Signal-to-cutoff ratio
RUO: Research use only
LoD: Limit of detection
IU/mL: International units per milliliter
Ct: Cycle threshold
SPSS: Statistical Package for the Social Sciences
WHO: World Health Organization

## References

[1] World Health Organization. Global progress report on HIV, viral hepatitis and sexually transmitted infections, 2021: accountability for the global health sector strategies 2016–2021: actions for impact. Geneva: World Health Organization; 2021.

[2] Rosen HR. Clinical practice. Chronic hepatitis C infection.N Engl. J Med. 2011;364:2429–38. 10.1056/NEJMcp1006613 21696309

[3] Türk Karaciğer Araştırmaları Derneği; Viral Hepatitle Savaşım Derneği. Türkiye hepatit C tanı ve tedavi kılavuzu—2023. 2023. Accessed December 21, 2024. Available at: https://www.tkad.org.tr/wp-content/uploads/2023/11/TURKIYE-HEPATIT-C-TANI-VE-TEDAVI-KLAVUZU-2023.pdf.

[4] Falade-Nwulia O, Suarez-Cuervo C, Nelson DR, et al. Oral direct-acting agent therapy for hepatitis C virus infection: a systematic review. Ann Intern Med. 2017;166(9):637–48. 28319996 10.7326/M16-2575 PMC5486987

[5] Roger S, Ducancelle A, Le Guillou-Guillemette H, Gaudy C, Lunel F. HCV virology and diagnosis. Clin Res Hepatol Gastroenterol. 2021;45(3):101626. 33636428 10.1016/j.clinre.2021.101626

[6] Baber AS, Suganthan B, Ramasamy RP. Current advances in hepatitis C diagnostics. J Biol Eng. 2024;18:48. 39252065 10.1186/s13036-024-00443-2 PMC11385151

[7] Tao Y, Tang W, Fajardo E, et al. Reflex hepatitis C virus viral load testing following an initial positive hepatitis C virus antibody test: a global systematic review and meta-analysis. Clin Infect Dis. 2023;77:1137–56. 37648655 10.1093/cid/ciad126

[8] Manuylov VA, Gushchin VA, Chulanov VP, et al. Regional, age, and sex patterns of hepatitis C virus infection in Russia: insights from a 42,000-participant serosurvey. Viruses. 2025;17:1529. 10.3390/v17121529. PMC12737774 41472200

[9] Ghany MG, Ward JW, Baldwin Z, et al. HCV testing and treatment of adults in the United States: 2014 through 2021—data from two national commercial testing laboratories. J Viral Hepat. 2025;32:e70087. 10.1111/jvh.70087. 41017733 PMC12477661

[10] Minissale MG, Petta S, Cartabellotta F. HCV screening in a Sicilian center: a descriptive cohort. profile Viruses. 2025;17:1252. 10.3390/v17091252. 41012678 PMC12474145

[11] López-Martínez R, Arias-García A, Rodríguez-Algarra F, et al. Significant improvement in diagnosis of hepatitis C virus infection by a one-step strategy in a central laboratory: an optimal tool for hepatitis C elimination?J. Clin Microbiol. 2019;58:e01815–19. 10.1128/JCM.01815-19 PMC6935937 31694971

[12] Bartlett SR, Yu A, Chapinal N, et al. The population level care cascade for hepatitis C in British Columbia, Canada, as of 2018: impact of direct-acting antivirals. Liver Int. 2019;39:2261–72. 31444846 10.1111/liv.14227

[13] World Health Organization. Global health sector strategies on HIV, viral hepatitis and sexually transmitted infections for the period 2022–2030. Geneva: World Health Organization; 2022.

[14] Johnson LN, Gaynor AM, Wroblewski K, Buss SN. Maximizing reflexive hepatitis C virus RNA testing of HCV antibody-reactive samples within United States public health laboratories. J Infect Dis. 2024;229(Suppl 3):S357–61. 37739790 10.1093/infdis/jiad191 PMC11078303

[15] Chen SH, Ding YJ, Hsu NT, et al. Prevalence-guided anti-HCV and reflex HCV antigen testing in the detection of patients with chronic hepatitis C in hepatitis C endemic areas. Diagnostics. 2025;15:3064. 10.3390/diagnostics15233064. PMC12691952 41374445

[16] Lee MK, Alfego D, Walworth C, Gillim L. Trends in reflexive HCV testing in a national US laboratory: a retrospective study from 2018–2024. Clinical Infectious Diseases. 2026:ciag227. 10.1093/cid/ciag227. 41964422

[17] Kirişci Ö, Calıskan A. Threshold value of the anti-HCV test in the diagnosis of HCV infection.J Infect. Dev Ctries. 2019;13:914–9. 10.3855/jidc.11657. 32084022

[18] Ocal M, Bulut ME. Determination of anti-HCV signal-to-cut-off value in patients with hepatitis C virus infection and the variety of antibody responses. Eur Res J. 2023;9:484–94. 10.18621/eurj.945588.

[19] Al Rawashdh N, Ferrufino CP, Kahn EB, et al. Cost-effectiveness of point-of-care hepatitis C virus RNA testing in the United States. JAMA Netw Open. 2026;9:e262658. 10.1001/jamanetworkopen.2026.2658. 41860547 PMC13005159

