# Supplementary material for: Missed confirmatory HCV RNA testing in anti-HCV-reactive patients and the role of reflex testing in improving completion of the hepatitis C diagnostic care cascade: a laboratory-based retrospective study
Source: BMC Infect Dis. 2026 Jun 19;26:1584. doi: 10.1186/s12879-026-13807-4 (PMC13528171; doi:10.1186/s12879-026-13807-4)
Supplement: Supplementary file 1 [file 12879_2026_13807_MOESM1_ESM.docx]

**Supplementary Table S1A**. Frequency of repeated anti-HCV testing during the study period and characteristics of patients undergoing frequent repeat testing

| **Number of**  **anti-HCV tests** | **Number of patients (n)** | **Percentage among repeatedly tested**  **patients (%)** | **Main clinical settings** |
| --- | --- | --- | --- |
| 2 | 2,484 | 79.1 | Various outpatient and inpatient clinics |
| 3 | 468 | 14.9 | Various outpatient and inpatient clinics |
| 4 | 113 | 3.6 | Various outpatient and inpatient clinics |
| 5 | 45 | 1.4 | Various outpatient and inpatient clinics |
| ≥6 | 30 | 1.0 | Hematology, bone marrow transplantation, general surgery/transplantation, nephrology |
