# Supplementary material for: Missed confirmatory HCV RNA testing in anti-HCV-reactive patients and the role of reflex testing in improving completion of the hepatitis C diagnostic care cascade: a laboratory-based retrospective study
Source: BMC Infect Dis. 2026 Jun 19;26:1584. doi: 10.1186/s12879-026-13807-4 (PMC13528171; doi:10.1186/s12879-026-13807-4)
Supplement: Supplementary file 2 [file 12879_2026_13807_MOESM2_ESM.docx]

**Supplementary Table S1B.** Clinical characteristics of patients undergoing frequent repeated anti-HCV testing (≥6 tests)

| **Clinical setting** | **Number of patients (n)** |
| --- | --- |
| Bone marrow transplantation / hematology-related follow-up | 22 |
| General surgery / transplantation clinic | 3 |
| Employee health unit | 3 |
| Hemodialysis service / nephrology-related follow-up | 1 |
| Urology service | 1 |
| **Total** | **30** |
