# Supplementary material for: Missed confirmatory HCV RNA testing in anti-HCV-reactive patients and the role of reflex testing in improving completion of the hepatitis C diagnostic care cascade: a laboratory-based retrospective study
Source: BMC Infect Dis. 2026 Jun 19;26:1584. doi: 10.1186/s12879-026-13807-4 (PMC13528171; doi:10.1186/s12879-026-13807-4)
Supplement: Supplementary file 3 [file 12879_2026_13807_MOESM3_ESM.docx]

Supplementary Figure S1. Distribution of anti-HCV signal-to-cutoff (S/CO) ratios according to HCV RNA status. Anti-HCV S/CO ratios were compared between HCV RNA-negative and HCV RNA-positive patients. HCV RNA-positive cases included both patients identified through routine confirmatory testing and those identified through retrospective testing without an initial clinician request. Similarly, HCV RNA-negative cases included both routinely tested and retrospectively tested patients in the pooled analysis. The figure illustrates the distribution of anti-HCV S/CO ratios according to HCV RNA status.
